# Triple Space-Time Yield in Discontinuous Antibody Biomanufacturing by Combination of Synergetic Process Intensification Strategies

**DOI:** 10.3390/bioengineering10121391

**Published:** 2023-12-05

**Authors:** Lucas Nik Reger, Martin Saballus, Markus Kampmann, Rene H. Wijffels, Dirk E. Martens, Julia Niemann

**Affiliations:** 1 Corporate Research, Sartorius, 37079 Göttingen, Germany; martin.saballus@sartorius.com (M.S.); markus.kampmann@sartorius.com (M.K.);; 2 Bioprocess Engineering, Wageningen University, 6708 PB Wageningen, The Netherlands; rene.wijffels@wur.nl (R.H.W.); dirk.martens@wur.nl (D.E.M.)

**Keywords:** CHO cell culture, process intensification, fluidized bed centrifuge, monoclonal antibodies, intermediate harvest, discontinuous biomanufacturing

## Abstract

Monoclonal antibodies are the workhorse of the pharmaceutical industry due to their potential to treat a variety of different diseases while providing high specificity and efficiency. As a consequence, a variety of production processes have been established within the biomanufacturing industry. However, the rapidly increasing demand for therapeutic molecules amid the recent COVID-19 pandemic demonstrated that there still is a clear need to establish novel, highly productive, and flexible production processes. Within this work, we designed a novel discontinuous process by combining two intensification strategies, thus increasing inoculation density and media exchange via a fluidized bed centrifuge, to fulfill the need for a flexible and highly productive production process for therapeutic molecules. To establish this new process, firstly, a small-scale experiment was conducted to verify synergies between both intensification strategies, followed by a process transfer towards the proof-of-concept scale. The combination of these two-process intensification measures revealed overall synergies resulting in decreased process duration (−37%) and strongly enhanced product formation (+116%) in comparison to the not-intensified standard operation. This led to an impressive threefold increase in space-time yield, while only negligible differences in product quality could be observed. Overall, this novel process not only increases the ways to react to emergency situations thanks to its flexibility and possible short development times, but also represents a possible alternative to the current established processes due to high increases in productivity, in comparison to standard fed-batch operations.

## 1. Introduction

In recent years, the biopharmaceutical industry has received much attention due to the unprecedented COVID-19 pandemic [1]. A key factor in fighting the pandemic was the increased speed of development of novel vaccines and drug treatments against COVID-19 [2,3,4]. However, the recent pandemic revealed further drawbacks, mainly current therapeutic molecule manufacturing, which lacks highly flexible, productive, and robust processes that can be rolled out across production sites worldwide in a short time frame [5,6]. Interestingly, this type of process is not only lacking in terms of the rare case of a global pandemic, but also in ordinary biopharmaceutical production, which is facing an increasing quantity demand for pharmaceutical products and an overall increase in protein-based therapeutics [7,8]. As a consequence, new and intensified production processes are needed that are flexible and easy to implement, have high volumetric productivities, and allow for efficient plant utilization. In this context, mAb production is suitable as a model system due to the existing platform processes as well as the long history of application.

mAb-based products are generally produced utilizing mammalian cell lines like Chinese hamster ovary (CHO) cells. This is due to their unique ability to generate appropriate post-translational modifications for human applications, their status as a safe host, and their well-known scale-up characteristics [9]. Currently, established production operations like fed-batch (FB) and perfusion formats are applied for the industrial production of mAb products. Both process formats have their own advantages and disadvantages with respect to biopharmaceutical production. Perfusion processes have a long cultivation duration and high volumetric productivity; however, the development of perfusion processes takes a relatively long time and many resources, in addition to requiring more complex process control. These drawbacks make them unsuitable for emergency situations, like pandemics, where there is a need for rapid development and quick process transfer to global production facilities [10]. FB formats, on the other hand, have a short cultivation duration and lower development efforts in terms of time and resources, which allows for rapid process development and integration into existing production facilities. However, they have comparatively low volumetric productivity, which is caused by their low peak cell density, long initial growth phase, and increased down time; these factors limit their potential to meet global needs rapidly [11,12]. To tackle the productivity limitations of FB processes, different process intensification strategies have been established over the last few decades to increase process yields. Early approaches mainly focused on modification of the standard process parameters during the late stage of cultivation by applying, for example, a temperature or pH shift. This shift generally resulted in improved cell viabilities and extended production times, and, thus, higher mAb yields [13,14]. Another intensification strategy is based on the addition of specific compounds to enhance productivity within the late processing phase. Supplements of carboxylic acids, such as natrium butyrate and valeric acid, have been shown to strongly improve productivity [6,15]. With the perfusion processes becoming more robust, firstly, combinations of both process types were investigated. One such strategy is the process established by Hiller et al. [16], which integrated a perfusion operation in the exponential phase of a FB process, resulting in enhanced cell densities and, consequently, an increase in volumetric productivity. Another example is the so-called high-inoculation fed-batch (HiFB), which is an FB that starts at a high cell concentration by using an increased cell density inoculum from a high-cell-density perfusion seed train (*n* − 1 perfusion). In this way, the low-cell-density growth phase of the fed-batch is moved from the large production reactor to a much smaller perfusion system. The intensification by HiFB reaches similar cell densities to the common FB, and, therefore, yields similar final product titers [6,17,18]. The main advantage of this process is that it shortens the duration of each cultivation and thus increases the number of runs per year, as well as the capacity of the production facility. To further enhance the output of a standard FB process, our group recently developed a new intensification method, the so-called Intermediate Harvest (IH), comprising a gentle and rapid media exchange within the exponential growth phase of the process [19,20]. This process did not alter the overall processing time but allowed for a strong increase in final product titers by significantly increasing viable cell densities. It is unknown whether this IH principle can be combined with the high starting cell densities of a high-inoculation fed-batch, which would result in high cell densities and shortened process duration, thus yielding even higher volumetric productivities. 

To rapidly respond to increases in global demands, production processes need to be intensified to give high yields in a short amount of time. The goal of this study was to investigate the potential of combining a high-inoculation FB (HiFB), defined by a comparably short process duration, with the Intermediate Harvest (IH) intensification strategy, characterized by increased viable cell density, to further enhance the overall productivity of the process that is referred to as the HiFB-IH process.

## 2. Materials and Methods

### 2.1. The Cell Culture System 

An industry-relevant Chinese hamster ovary cell line (CHO-DG44) stably expressing an IgG_1_ (pI: 7.3) product was used as a model system. For media, the chemically defined 4Cell^®^ SmartCHO system (Sartorius Stedim Biotech, Göttingen, Germany) was utilized for all cultivations from pre-culture up to the production reactors. To remain consistent, all pre-cultures were processed similarly. 

### 2.2. Small-Scale Cultivation

The Ambr15 (Sartorius Stedim Biotech, Göttingen, Germany) small-scale system was used to characterize the new HiFB-IH intensification compared to a standard fed-batch (std. FB) and a high-inoculation fed-batch using triplicate cultivations for all approaches. Possible scalability-relevant factors have been evaluated in other works [21]. Basic parameters for the bioreactors were: temperature of 36.8 °C; pH of 7.1, adjusted by CO_2_ sparging; and DO of 40% by additional O_2_ gassing and agitation at 1300 rpm. To mimic the n-1 perfusion, cells from a common batch seed train were concentrated by centrifugation (4-16KS, Sigma, Osterode am Harz, Germany) at 190× *g* for 3 min up to 100 × 10^6^ cells/mL. std. FB reactors were inoculated at 0.3 × 10^6^ cells/mL, while the high-inoculation fed-batches were inoculated at 5 × 10^6^ cells/mL [6]. All bioreactors of the std. FB and HiFB were fed as described in a previous work [6]. HiFB-IH vessels were operated in a similar way to the HiFB process up to day 2.5, at which point a media exchange was conducted [19]. In short, each vessel was removed from the system and centrifuged at 190× *g* for 5 min, the medium was removed, and a fresh enriched basal medium was supplied to the vessel [22]. To account for the increased cell densities due to the HiFB-IH approach, a flexible feeding scheme was applied as stated in our previous work [23]. Briefly, the feeding scheme was based on a multiplication factor calculated by the daily viable cell volume (VCV) divided by the peak VCV of a std. FB process (34.9 mm^3^/mL). The amount of feed to be added was divided over two additions per day (in 12 h intervals) to prevent single-point overfeeding. The process was stopped when the viability dropped below 70%. 

### 2.3. Proof of Concept

**Figure 1:**
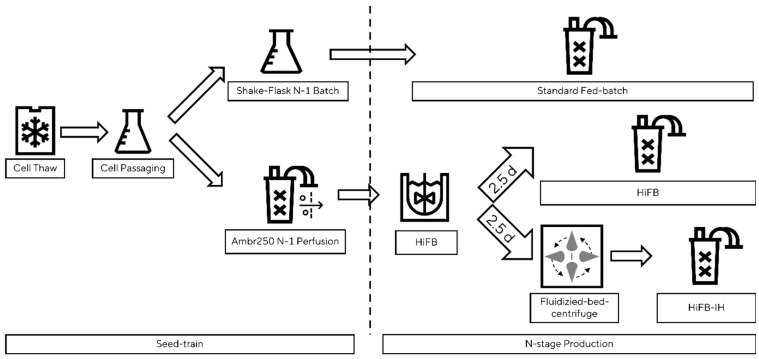
Schematic overview of proof-of-concept scale cultivation, including the fluidized bed centrifuge. Figure shows the separation, starting from the seed train until the three n-stage process, including a standard fed-batch, a high-inoculation fed-batch, and a high-inoculation fed-batch coupled with an intermediate harvest step.

A schematic presentation of the benchtop set-up is shown in Figure 1. To showcase the scalability of the newly designed process, the std. FB, HiFB, and HiFB-IH were cultivated in benchtop bioreactors in at least duplicate experiments. For the std. FB, the common pre-culture scheme was utilized, while the seed train for the HiFB and HiFB-IH was conducted in a perfusion system (Ambr250Perfusion (Sartorius Stedim Biotech, Göttingen, Germany) to generate enough cells for high-cell-density inoculation. An ATF-based single-use vessel was inoculated with 0.2 × 10^6^ cells/mL, and after a 3-day batch phase, the media exchange via the filter system was started. In each cultivation phase, a CSPR of 50 pg/c/d was maintained. Enriched basal media were used as the perfusion medium. After the pre-culture perfusion, a 5 L UniVessel (Sartorius Stedim Biotech, Göttingen, Germany) was inoculated with 5 × 10^6^ cells/mL at a 3 L working volume. After 2.5 days, the cell broth was split in half and transferred into an Ambr250 system (Sartorius Stedim Biotech, Göttingen, Germany) to compare the new HiFB-IH method to the HiFB reference method using cells from the same pre-culture. The cell broth for the HiFB references was directly transferred to the Ambr250 vessel, whereas for the HiFB-IH method, the cell broth was washed and concentrated using fluidized bed centrifugation (see Section 2.4) prior to the transfer to the Ambr250. Similar to the small-scale process, a VCV-dependent feeding for the HiFB-IH was utilized, divided over two addition moments per day. The std. FB culture was conducted in an Ambr250 vessel following the platform process, as described in [6]. All cultivations within a group (std. FB, HiFB, HiFB-IH) were finished after the threshold of 70% viability was reached in one of the replicates.

### 2.4. Cell Wash and Concentration

Aiming for a scalable single-use operation for separation, washing, and concentration of cells, a Ksep^®^ 400 fluidized bed centrifugation (FBC) system (Sartorius Stedim Biotech, Göttingen, Germany) was used. The cell broth was sterile, and was processed with the system’s cell wash harvest mode using the appropriate consumables and parameters according to a previous work [23]. 

In brief, a cell broth volume of 1750 mL derived from the benchtop bioreactor was loaded, during a single process cycle, into the FBC. The cells were captured, concentrated, and washed with 37 °C tempered phosphate-buffered saline (PBS, all chemicals supplied by Carl Roth, Karlsruhe, Germany). Cells concentrated in 300 mL PBS were transferred to an intermediate vessel in which approximately 250 mL of temperature-controlled enriched basal medium had already been prepared to avoid nutrient limitation during the IH step. The VCC was determined, then adjusted further by enriched basal media. Next, the diluted cell broth was transferred into two Ambr250 culture vessels.

### 2.5. Analytics

For all cultivations, samples were taken prior to each feeding, and for the HiFB and HiFB-IH cultivations, an extra sample after the feed at day 2.5 was taken. The CEDEX HiRes (Roche, Basel, Switzerland) was utilized to measure cell concentration and viability by trypan blue exclusion and cell diameter. pH and metabolite (glucose and lactate) concentrations were measured with an ABL800 basic radiometer. Samples for further analysis were centrifuged at 6600× *g* for 5 min, and the supernatant was stored at −20 °C. 

To quantify the antibody concentrations over time, size exclusion chromatography (SEC) was utilized. All measurements were made with the Vanquish™ Flex UHPLC System (Thermo Scientific, Waltham, MA, USA) equipped with a Yarra™ 3 μm SEC-3000 (Phenomenex, Torrance, CA, USA). The flow rate was set to 1 mL/min, and a specific antibody std. curve was prepared. 

The N-glycosylation of each approach on the harvest day was determined by utilizing the LabChip^®^ GXII Touch™ HT Protein Characterization System (PerkinElmer, Waltham, MA, USA). Prior to the determination, each sample was purified via protein A (PreDictor MabSelect SuRe 96-well plates from Cytivia, Marlborough, MA, USA) and desalted using Vivaspin 500 Centrifugal Concentrators (Sartorius Stedim Biotech, Göttingen, Germany) with a molecular weight cutoff of 10 kDa. Subsequently, each sample was prepared as described in the manual by the Glycan Release and Labeling Kit (Perkin Elmer, Waltham, MA, USA), then measured. 

The specific productivity (Qp) and integral of viable cell concentration (IVCC) were calculated as stated in the previous work [23]. Cell-based volumetric productivity (Qp_c_) and the integral of viable cell volume (IVCV) were calculated similar to the corresponding Qp and IVCC values. Further, all equations are provided within the Supplementary Materials (Equations (S1)–(S5)).

## 3. Results

The current work aimed to combine two intensification strategies, i.e., the high-inoculation FB (HiFB), resulting in comparable titers in less time, and the Intermediate Harvest (IH) concept, resulting in higher titers in the same amount of time. The newly designed process will be referred to as high-inoculation fed-batch coupled with Intermediate Harvest (HiFB-IH). First, a small-scale cultivation was performed, where the HiFB-IH process was compared to the standard FB (std. FB) and HiFB, to provide insight into the potential of the process. Subsequently, the scalability of the novel process could be confirmed in a proof-of-concept study.

### 3.1. Small-Scale Experiments

**Figure 2:**
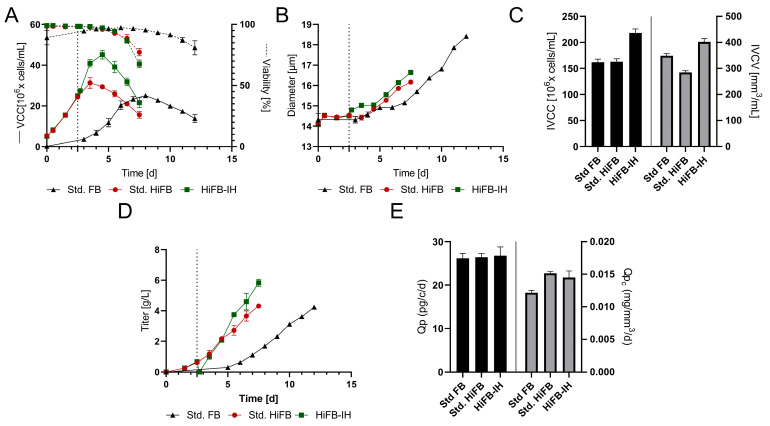
Performance of the HiFB-IH process compared to the standard FB and HiFB processes on a 15 mL scale. The media exchange is marked by dotted lines for the HiFB-IH process. (**A**) Viable cell count (VCC) and viability over the course of cultivation. (**B**) Diameter changes within the timeframe. (**C**) Calculated values for the integral of viable cell count (IVCC) and integral of viable cell volume (IVCV). (**D**) Titer expression over the course of cultivation for all process formats. (**E**) Cell-specific productivity (Qp) and cell-volume specific productivity for the three processes.

To obtain a first impression on the feasibility and characteristics of the new process, a small-scale experiment was performed comparing the new HiFB-IH with a std. FB process and an HiFB process. The viable cell densities (VCC) are shown in Figure 2A, revealing typical growth progression for the chosen cell line in a std. FB with 25 × 10^6^ cells/mL peak cell density and an overall duration of 12 days until reaching the viability threshold of 70%. The HiFB shows increased VCC values in the first days compared to the std. FB, caused by the higher inoculation density of 5 × 10^6^ cells/mL compared to 0.3 × 10^6^ cells/mL (std. FB). Furthermore, a small increase in peak cell density at day 3.5 up to 31 × 10^6^ cells/mL was observed. The growth for the HiFB-IH was similar to that of the HiFB up to day 2.5, as expected. At day 2.5, the media exchange was conducted for the HiFB-IH, resulting in a subsequent high increase in peak cell density up to 45.15 × 10^6^ cells/mL, an increase of around 45% compared to HiFB and 80% compared to std. FB. The viability for both high-inoculation process types showed a comparable decrease in viability (<70%) up to day 7.5, 4.5 days earlier than the std. FB. Another important parameter is the cell diameter. As can be seen from the data displayed in Figure 2B, the initial cell diameter was the same for all cultures, with a value of around 14 µm, which can be explained by the fact that all cultures were inoculated from similar seed train cultures and only differed by a centrifugation step, as described in the Methods section (Section 2.2). Thereby, the HiFB-IH cultures showed an initial small increase in diameter at day 2.5, overlapping with the media exchange, and subsequently, all other cultures showed enhancing diameters starting at day 4. At day 7.5, marking the harvest day for the HiFB and HiFB-IH, the cell diameters of the HiFB (16 µm) and HiFB-IH (16.5 µm) showed enhanced values compared to the std. FB (15.5 µm). However, after day 7.5, the std. FB showed a further increase in diameter up to 18.4 µm on the final cultivation day, day 12. Next, the integrals of the viable cell concentration (IVCC) and the viable cell volume (IVCV) were calculated for all three culture types and are shown in Figure 2C. The IVCC showed an increased value for the HiFB-IH (+33%) and, as expected, comparable values for the HiFB and std. FB. For the IVCV, which included changes in diameter, the value for the HiFB-IH was still the highest, substantially higher (41.5%) than that of the HiFB. However, an overall decrease for the IVCV compared to the IVCC values was observed for both the HiFB (−18.3%) and HiFB-IH (+15.5%) cultures in contrast to the std. FB process, possibly caused by the greater increase in diameter for the std. FB process. 

Figure 2D shows the titers of the different processes as functions of time. Similar final titers of around 4.3 ± 0.06 g/L were found for the std. FB and the HiFB. The HiFB-IH showed a similar trend as the HiFB, up to 0.6 ± 0.01 g/L at day 2.5, when the medium was replaced, causing a strong reduction in titer. Subsequently, a faster increase in the mAb concentration was observed for the HiFB-IH process, up to 5.8 ± 0.18 g/L at day 7.5. Including the mAb produced in the first 2.5 days before the medium exchange, this approach produced around 6.4 g/L, 50% more than both other processes. To further study these differences, the specific productivity was calculated on a per-cell and per-cell-volume basis (Figure 2E). For the specific productivity per cell, similar values of around 26 pg/c/d were found for all processes. Since cells in the std. FB reached a higher volume than those in the other two processes, the cell volume-based specific productivity was lower in the std. FB than in the other two processes. In summary, the small-scale trial showed the expected values for the std. FB and HiFB, reaching comparable titers, but in a shorter time for the HiFB. Meanwhile, the HiFB-IH reached higher titers, possibly due to the increased IVCC values in the same amount of time as the HiFB, and, thus, in a shorter time than the std. FB. This leads to the further preliminary conclusion that a combination of both process intensifications could adopt both beneficial characteristics and show increased cell densities and final titers. Therefore, the basic feasibility was shown, and the next step was to demonstrate scalability on a benchtop scale using a fluidized bed centrifuge. 

### 3.2. Proof of Concept

To show a full proof of concept, the newly developed process was scaled up, and a fluidized bed centrifuge (FBC) was incorporated into the process. The complete process scheme is described in Section 2.3. Similar to the small-scale cultivation, all three different approaches (std. FB, HiFB, HiFB-IH) were studied in at least duplicate. 

**Figure 3:**
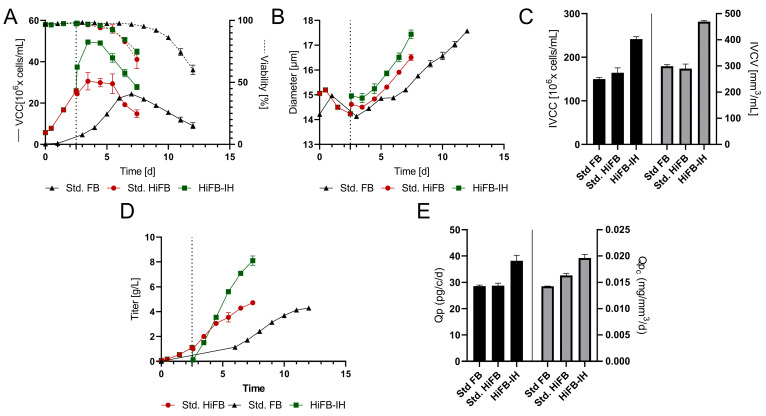
Proof of concept (PoC) of the new designed process in fully scalable process systems. Standard fed-batch (standard FB) was cultivated fully on a 250 mL scale. The high-inoculation variants (high-inoculation fed-batch (standard HiFB and high-inoculation fed-batch coupled with Intermediate Harvest (HiFB-IH)) were first cultivated at the 5 L scale and transferred at day 2.5 to the 250 mL scale. Dotted lines represent the Intermediate Harvest at specified points for the HiFB-IH. (**A**) Viable cell counts and viability over the cultivation period. (**B**) Diameter variation for all three processes within the PoC. (**C**) Integral of viable cell count (IVCC) and integral of viable cell volume (IVCV) over the complete cultivation period. (**D**) Concentration of expressed mAb for all process formats. (**E**) Expression characteristics in the form of cell-specific productivity (Qp) and cell-volume specific productivity (Qp_c_) for all process formats.

Figure 3A displays the VCC and viability for all processes, revealing similar growth, peak cell density, and cultivation duration for the std. FB (24 × 10^6^ cells/mL; 12 days) and the HiFB (30 × 10^6^ cells/mL; 7.5 days) compared to the small-scale cultivation. Meanwhile, the HiFB-IH revealed small alterations to the small-scale experiment, but still showed a similar duration (7.5 days) and comparable peak cell density (49 × 10^6^ cells/mL). The alteration occurred mainly around day 2.5 of the HiFB-IH cultivation, showing a strong increase in cell concentration directly after the medium exchange, which was not present at the small scale. This increase in cell count for the HiFB-IH approach was caused by the FBC system, which concentrated the cells and subsequently resuspended the cells in less volume, which is not possible at the small scale (further discussed in Section 4.1). For further insight into the process, the cellular diameter is shown in Figure 3B, revealing deviated values from the small scale for the HiFB and HiFB-IH processes (Figure 3B). An increased cellular diameter (+0.8 µm) after inoculation was visible for the high-inoculation-density approaches, likely resulting from the different seed trains utilized, i.e., *n* − 1 perfusion for the intensified process and *n* − 1 batch for the std. FB cultures. Furthermore, an enhanced increase in diameter over time could be observed for the high-inoculation approaches as compared to the small scale, especially for the HiFB-IH, resulting in only a small difference at the end of each cultivation of −2 µm (HiFB) and −0.1 µm (HiFB-IH) compared to the std. FB. Further, the IVCC and IVCV values were calculated and can be seen in Figure 3C. A small increase in the IVCC for the HiFB (+10%) and a strong increase for the HiFB-IH (+61%) could be observed in comparison to the std. FB display, with a high level of similarity to the small-scale. Meanwhile, the IVCV showed a variated picture compared to the small scale, with a similar biomass volume for the HiFB and a high increase in the HiFB-IH (+57%) compared to the std. FB. This alteration may possibly be caused by the variated diameter trend between the small-scale and the benchtop reactor. 

Another distinction from the small-scale experiment is visible for the mAb concentration in Figure 3D. While only minor differences were observed between the std. FB (4.2 ± 0.06 g/L) and the HiFB (4.7 ± 0.12 g/L), as is comparable to the small-scale, the HiFB-IH showed higher titer results at the benchtop scale than at the small scale, reaching 1.1 g/L in the first cultivation phase (up to day 2.5) and 8.1 ± 0.3 g/L in the second phase of the process. This resulted in an overall production of 9.2 ± 0.3 g/L in 7.5 days. To further assess the process-related characteristics, the cell-specific productivities (Qp) were calculated on a per-cell and per-cell-volume basis and are shown in Figure 3F. The specific productivity per cell showed similar values for the HiFB and the std. FB as in the small-scale experiments. However, a significant increase of around 35% compared to both other approaches was found for the HiFB-IH, which was different from the small scale. This difference was also observed when expressing the productivity per cell volume (Qp_c_), revealing a +38% increase for the HiFB-IH in comparison to the std. FB. Further, a small increase for the Qp_c_ could be identified for the HiFB (+14.7%) compared to the std. FB, which ranged between those for the other processes. In summary, at a benchtop scale using an FBC, it was shown that high inoculation can successfully be combined with Intermediate Harvest, resulting in a shorter processing time (compared to std. FB) and higher titers. 

### 3.3. Space-Time Yield and Glycosylation

**Figure 4:**
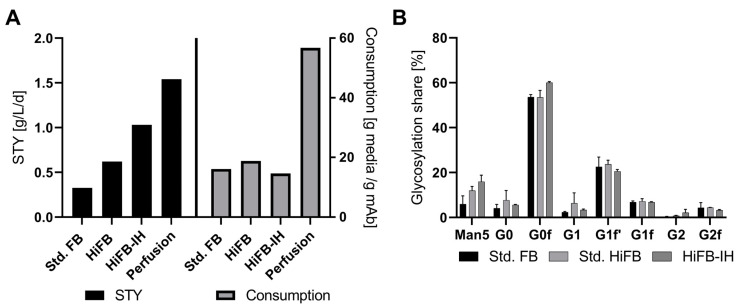
(**A**) Performance attributes in the form of space-time yield (STY), in g/L/d, for the three processes and the perfusion application, as well as the consumption of media per produced antibody (g media/g mAb). (**B**) Generated data regarding critical quality attributes (CQA) in the form of glycan distribution for the produced mAb of all three compared process types: standard fed-batch (standard FB), high-inoculation fed-batch (standard HiFB), and high inoculation coupled with Intermediate Harvest (HiFB-IH).

To further compare the processes on an economic level, performance attributes like the space-time yield (STY), revealing the production in g per L of bioreactor volume per time period, and the media consumption in gram media per gram of produced mAb, were calculated. For this, the perfusion process was included as a benchmark of high productivity, where the respective data originated from a similar clone to that used in the present study [23]. In short, duplicate perfusion cultivation in a 250 mL reactor system with a filter cell retention device was performed. Standard parameters were fixed at 2.5 volume exchanges per day and 50 × 10^6^ cells /mL as the cell density target. Figure 4A shows the respective STY values in ascending order, with the std. FB at 0.325 g/L/d followed by the HiFB (0.62 g/L/d) and HiFB-IH (1.03 g/L/d), revealing a threefold increase in the combined process intensifications, while the perfusion process had an even higher value (1.54 g/L/d). Therefore, the HiFB-IH allows for a significant reduction in the productivity gap that can be observed between discontinuous processes and continuous perfusion cultivation, with only around 33% less productivity on a daily basis. Furthermore, in Figure 4A, on the right side, the utilized amount of media powder per gram of produced antibody is shown. To compare all processes, 5 L UV cultivation was used as the standard, and the pre-culture medium was incorporated into the calculation. As can be seen, the three discontinuous process types utilized comparable amounts of media per unit antibody, with the lowest used for the HiFB-IH at 14.8 g/g, revealing a slight decrease in media consumption of −1.3 g/g compared to the std. FB and −4 g/g in comparison to the HiFB. In contrast, the steady-perfusion process consumed significantly more media to produce a defined amount of product, with around 56 g/g, representing a 378% increase compared to the HiFB-IH, caused by the continuous character of this cultivation. In addition to the cellular performance and expression-related characteristics, the quality of the expressed antibody also needs to be considered with respect to its potency and possible side effects. Therefore, to compare the quality of the produced mAb product, the glycan structure for each process was determined. In Figure 4B, the eight measured glycan structures are shown, revealing an increased share of the Man5 structure towards the higher intensified process types like HiFB (+6%) and HiFB-IH (+10%). In addition, comparable structures were observed without any noticeable deviations between any of the tested process types. 

In summary, our data show a successful combination of two promising process intensification strategies: the high-inoculation fed-batch (HiFB) and the Intermediate Harvest (IH) approach. Therefore, the novel HiFB-IH process allowed for a threefold increase in volumetric productivity of up to 1 g/L/d, even with slightly decreasing media consumption rates (media/product), while only minor glycan quality deviations were present. Furthermore, our work shows an easy-to-adapt, small-scale system for the fast and reliable development of this new process type. 

## 4. Discussion

The aim of this study was to design a process characterized by (i) high volumetric product yields to respond to global needs, (ii) easy implementation in different existing production facilities, and (iii) short development times enabled by robust downscale models. Therefore, two promising process intensification strategies were combined in a conventional, discontinuous FB process: a high-inoculation fed-batch (HiFB) strategy to shorten the overall cultivation time and a novel media exchange strategy (IH) to increase the overall cell density as well as cell-specific product expression. The novel process design was developed using a 15 mL small-scale system, then subsequently transferred into a proof-of-concept scale comprising a media exchange via a fluidized bed centrifuge. 

### 4.1. Process Characterization and Comparison

The novel HiFB-IH investigated in this work combines two established process intensification strategies, the high-inoculation fed-batch (HiFB) and the Intermediate Harvest (IH). Following the HiFB strategy, the process was inoculated at 5 × 10^6^ cells/mL, which represents a considerably higher cell density than that for a standard FB (std. FB) process (compare [6]). As a result, the low-productivity phase occurring during the initial growth phase of the std. fed-batch process (day 0–3) was bypassed (Figure 2A and Figure 3A). This led to an overall shorter process time. The product yields at the end of the process were comparable to the std. FB, a distinct benefit that has been demonstrated in previous studies by our group and others [6,18,24]. However, while this intensification measure did increase the space-time yield of the process, it either failed to influence or only slightly influenced the peak cell densities and cell-specific productivities. Accordingly, we investigated the combination through a recently developed process intensification strategy called Intermediate Harvest (IH). This approach is based on a single complete media exchange at a defined process stage. Previous studies, not only from our group, have proven a significant increase in viable cell densities and overall volumetric productivity if a media exchange, either in a rapid fashion (IH) or via continuous perfusion, is performed during the exponential growth phase of the cells [16,19,23]. Due to the accelerated growth profile of the HiFB in comparison to the std. FB, day 2.5 was chosen for the IH to allow for a media exchange during the exponential growth phase of the process. In agreement with previous studies, further proliferation of the cells could be observed, with a strong increase in cell densities consecutive to the IH (+12 × 10^6^ cells/mL). This showed an increased trend compared to the HiFB culture without media exchange (+6 × 10^6^ cells/mL) from day 2.5 to 3.5 (Figure 3A). In addition to the further proliferation of the cells from day 2.5 to 3.5, the benchtop cultivation showed a significant increase in cell density (+12 × 10^6^ cells/mL) at day 2.5, as assessed by a comparison of the cell counts before and after utilizing the IH method. This increase in cell density was impacted by the volume reduction in the reactor supernatant by the FBC system, leading to a concentration of the cells similar to previous studies. The volume reduction within the process can be used to further boost the cell counts, and subsequently the productivity of the process, as has been shown in previous studies [19]. To utilize this method, the production reactor was inoculated with an increased starting volume to enhance the total amount of cells in the reactor in the first phase of the cultivation and reduce the volume at the IH step to enhance the cell counts. The concentration of cells was enabled by the functional principle of the utilized FBC, comprising a capture of the cells by simultaneous flowthrough of the small media and product components. Thereby, a concentration of cells up to 100 × 10^6^ cells/mL could be reached, as investigated in former studies [20]. This effect was also utilized for the current process combination between the HiFB and the IH, showing a high concentration of cells on the respective days. A possible drawback of this cell density increase due to volume reduction is the increase in inoculation cell densities or volumes (around +33%), which might require adjustments to the corresponding seed train. However, within common high-inoculation scenarios, perfusion seed trains are utilized, which should compensate for the overall effort expended for the increased cell densities, either due to increased cell densities or an increase in volume, to supply the n-stage production. 

Regarding the further course of HiFB-IH cultivation, no major differences in the cellular or viability profiles could be observed over time compared to the HiFB, except for generally higher cell densities of around +15 × 10^6^ cells/mL. This led to a similar final harvest day for both processes. This observation is in line with our previous studies, showing that an IH during the exponential phase does not affect the overall cultivation duration compared to the standard process (here, the HiFB). However, the overall increase in cell density leads to a logical boost in the IVCC and IVCV (Figure 3C), representing one reason for the enhanced titer values for this process in comparison to the std. FB (+116%) and HiFB (+95%). As a significant secondary impact factor in the titer increase, we were able to identify an increase in cell-specific productivity for the HiFB-IH process of around +58% (Qp) compared to the std. FB. A similar behavior has been detected in previous studies [19,23], and can be partially explained by the increase in diameter, as discussed within the previous work as well as other studies [25,26]. However, inclusion of the cellular diameter into the productivity calculation (Qp_c_) still showed an increase of 35% for the HiFB-IH process, indicating further underlying mechanisms for the increased product expression by the cells. Interestingly, this effect was not observed in the small-scale studies (Figure 2E), but could also be seen for previous IH in benchtop-scale studies [19,23]. One reason for the enhanced cell-specific productivity could be the complete media exchange within the FBC method in comparison to the small-scale, which showed residual spend media within the pellet [19]. This media exchange not only supplies new nutrients to the cells, but also decreases byproduct components which can possibly impact cellular expression. Another significant change between the scales was the use of the FBC system for media exchange in contrast to the small-scale experiment which used classical centrifugation methods. Here, the FBC system used both centrifugation and flow force to capture the cells, which differed significantly from classical centrifugation and may have led to the increased cell-specific productivities in the IH experiments due to differentiated stress input. A similar productivity increase was recently described by another research group, indicating that short periods of different levels of stress can enhance cellular expression [27]. Interestingly, other studies in the field of perfusion have shown a similar effect of enhanced cell-specific productivities for some cell lines, which can possibly be traced back to both aforementioned reasons, i.e., complete media exchange and mechanical stress within the filter-based cell retention devices [28,29]. The enhanced cell-specific productivities, possibly triggered by stress induction, seem to be an interesting factor and should be investigated in more depth to reveal possible desired intracellular changes.

Overall, the novel HiFB-IH confirms that a high-inoculation approach and the IH intensification strategy can be used in synergy to achieve short production durations with increased volumetric productivities, resulting in an threefold increase in STY compared to a common FB process.

### 4.2. Quality Assessments

In addition to the changes between the two scales, significant but minor changes in the CQAs of the produced antibody were detected for the HiFB-IH by analyzing the glycan composition in comparison to the two standard process types. The glycan share can influence the pharmacometrics of the produced antibody, affecting its potency as well as its toxicity profiles [30]. In general, the two mechanisms of action for mAb products, i.e., antibody-dependent cell-mediated cytotoxicity (ADCC) and complement-dependent cytotoxicity (CDC), can be influenced by the glycan composition. It has been shown that low-fucose structures can benefit ADCC, while a high galactosylation share can improve CDC [31,32]. Interestingly, the Man 5 glycan structures contain no core fucose motifs; therefore, a potential increase in ADCC effector function could be hypothesized as a result of the detected increase in Man5 content for the HiFB-IH process (compare Figure 4A). However, at the same time, this structure is a precursor for the galactosylated structure (G0-G2) [33]. A reasonable explanation for the increased Man 5 share for the HiFB-IH cultures could be an imbalanced supply of nutrients based on the IVCV feeding strategy. In support of this, increased osmolality values were detected in these cultivations, commonly associated with an increase in Man 5 structures [34]. To counteract the formation of Man 5 glycan structures, the feeding strategy was optimized to reduce the osmotic pressure on the cells. This not only decreased the Man5 glycan structures, but also optimized the media consumption caused by the process. 

### 4.3. Differences in Scales

Another goal of this work was to establish a small-scale screening system to rapidly set up a new process and screen different parameters. Therefore, the variated parameters within the scales will be discussed within this section to show the possible differences between the scales. One noticeable difference is visible throughout the IH method, revealing constant cell densities after the IH method within the small scale. Meanwhile, the proof-of-concept scale comprises a concentration up to around 38 × 10^6^ cells/mL after the media exchange. This difference is caused by the concentration step within the FBC, as is discussed in Section 4.1, and is not transferable to the small-scale system due to volume limitation. Another impacting factor is the liquid handling in the IH step in the small-scale process, leading to decreased recovery, which was observable in the previous work within this field as well [19,20]. These factors led to the small discrepancies for days 3.5 and 4.5 for the HiFB-IH in both scales; however, both cultivations realigned within the later phase and showed similar peak cell densities. Another factor impacting the small scale becomes visible by comparing the cell-specific productivity rates for both scales, which reveals decreased rates within the small scale. This finding is in line with our previous studies, and the increase in cell-specific productivities was discussed in Section 4.1. Overall, the two aforementioned variations impacted the small scale, leading to a decrease in overall titer yield within this cultivation. However, in our view, the applied small-scale cultivation still showed sufficient agreement with the scale-up data set, and, therefore, can be used as a suitable high-throughput process development tool. 

### 4.4. Economical Classification

The basic intent to establish process intensification strategies for new or existing platform processes is to decrease the footprint; increase the flexibility; and save time, and, most importantly, costs. A cost reduction when using the discontinuous format is commonly reached by increasing the STY of the process, either by boosting product titers while keeping the process duration constant or by decreasing the process duration while maintaining the same final titer. With our new process, we were able to combine both approaches and reach higher product titers in a shorter time frame. In order to further investigate the economic potential of the HiFB-IH, a comparison of the designed process with other applied processes like std. fed-batch and high-inoculation fed-batch was made. A two- to threefold increase in productivity compared to the two competitive discontinuous process types (HiFB and std. FB) was detected, caused by (i) the reduction in the cultivation duration, caused by the higher inoculation VCC of the HiFB-IH; (ii) the increased cell densities reached through the media exchange; and (iii) an increase in cell-specific productivities. Further, the newly developed process is able to draw on established platform processes in cell line development or production processes for discontinuous process formats to accelerate development. Moreover, the low adjustments needed to transition from a std. fed-batch to the new process could ease the implementation of the new process into existing production facilities, thus shortening and streamlining the transfer of technology into the production stage. In addition to the advantages of the HiFB-IH towards the other discontinuous process types, the strong increase in productivity allows for a comparison with a steady-state perfusion, revealing a 27% lower STY compared to the continuous bioprocess format. A key benefit of the discontinuous operation is the low media consumption rate throughout the process, as highlighted in Figure 4A, showing up to a 378% increase in media utilization for the continuous process. This is mainly caused by the continuous character of the steady perfusion, which demonstrates the necessity of keeping the cells within a proliferating state. One key improvement for the new process is the replacement enriched basal media by PBS used during the operation of the FBC for the IH method, further diminishing the media consumption of the FBC method. This switch in washing buffer towards PBS was tested in previous studies and showed no impact on the subsequent cell cultivation [23]. Further, discontinuous operations are commonly associated with decreased monitoring efforts due to the lower number of peripheral devices needed to stabilize the process [12,35]. This results in reduced labor costs compared to continuous operations that require extensive monitoring of the process itself, and, moreover, decreases the risk of possible system errors which would result in the termination of the cultivation. Notably, adding the FBC system and, therefore, an extra peripheral device will at least require some added monitoring effort. However, the FBC system is only utilized in short, defined timeframes, reducing the monitoring time to a minimum in comparison to perfusion via a constant exchange of media and associated monitoring and control. Another benefit of the HiFB-IH as a discontinuous process is the increased robustness and traceability of defined batches, alongside the higher flexibility [35]. These characteristics reduce risks around the manufacturing process that are not only related to low monitoring efforts, but also through clearly defined short-term batches. As an example, a failure of one batch within a year would only lead to a failure rate of around 2.7% (assumptions: 36 processes per year, duration 10 days); meanwhile, a failure of one batch for a steady-state perfusion would result in an 8.3% loss (assumptions: 12 processes annually, duration: 30 days). Furthermore, the traceability of defined batches can be an asset for GMP manufacturing, which is clearly given for the HiFB-IH, while for the perfusion process, no batch is explicitly defined [36,37]. Another benefit of the discontinuous process format is the enhanced flexibility due to the short, isolated batches [38]. This advantage can be further facilitated throughout the utilization of a single-use manufacturing system, which would allow for the production of several different products within one facility. However, this might increase the need for consumables, which is also associated with additional material costs, such as bioreactor bags in a single-use system, lines, or filters. Combined with this increase in consumables, the labor cost needed to assemble and prepare the system needs to be considered in comparison to a continuously operated system. Thereby, in parallel, the increased quantity of batches associated with the HiFB-IH and other discontinuous processes can also be considered disadvantageous, as it decreases plant utilization due to extended downtime in comparison to the steady perfusion process. However, to make further statements, a holistic cost analysis would be required that compares the different process formats, which was not the goal of this work. 

Overall, the novel process showed a set of advantages in comparison to the perfusion process. One imaginable use case could be the production of an emergency drug, for example, as a response to a pandemic. Thereby, the fast implementation of the process due to well-established platform processes for the FB operation, alongside existing small-scale screening tools, would be beneficial, as would the possibility of using existing single-use production plants constructed for FB cultivation. This could lead to a fast ramp-up of production capacity without the need to design new manufacturing suites. Furthermore, these plants could be utilized for the manufacturing of different drugs according to changing needs. In addition to emergency situations, applications within conventional production would also be conceivable due to the overall advantageous properties of the newly designed process, and could lead to a further reduction in the size of the plant due to increased productivity.

Finally, the novel HiFB-IH operation highlights the feasibility and potential of a rational combination of existing process intensification strategies to generate synergies and significantly boost productivity. 

## 5. Conclusions

The presented work describes the rational combination of two beneficial FB intensification strategies. We increased starting inoculation VCCs (HiFB) and introduced a media exchange via an FBC system (IH), resulting in a short-duration, highly productive, and discontinuous process. Compared to the benchmark FB process, the novel HiFB-IH strategy successfully combined the short cultivation duration achieved by the HiFB process (−37%) with the significantly increased cell densities (+61%) and product expression (+35%) characteristic of the IH process. Overall, these properties resulted in a threefold increase in space-time yield (STY), with nearly similar critical quality attributes to the standard FB. 

## Data Availability

The raw data supporting the conclusion of this article will be made available by the authors, without undue reservation.

## Supplementary Materials

The following supporting information can be downloaded at: https://www.mdpi.com/article/10.3390/bioengineering10121391/s1, Equation (S1): Calculation for integral of viable cell count (IVCC); Equation (S2): Calculation for the specific volume (VCV); Equation (S3): Integral of viable cell volume (IVCV) over the course of cultivation; Equation (S4): Calculation of the cell specific productivity (qP) over the course of cultivation; Equation (S5): Calculation of the cell specific productivity (Qpc) over the course of cultivation.
