# Supplementary material for: Triple Space-Time Yield in Discontinuous Antibody Biomanufacturing by Combination of Synergetic Process Intensification Strategies
Source: Bioengineering (Basel). 2023 Dec 5;10(12):1391. doi: 10.3390/bioengineering10121391 (PMC10740458; doi:10.3390/bioengineering10121391)
Supplement: Supplementary file 1 [file bioengineering-10-01391-s001.zip › bioengineering-2742246-supplementary.pdf]

Supplementary material

This document contain the supplementary data for the Manuscript "Triple Space-Time Yield in Discontinuous Antibody Biomanufacturing by Combination of Synergetic Process Intensification Strategies" by Lucas Nik Reger, Martin Saballus, Markus Kampman, Rene H. Wijffels, Dirk E. Martens, Julia Niemann.

The utilized equations for all calculations are stated within the first part of this supplementary.

The Integral of Viable cell count (IVCC) was by the following Equation S1 utilizing the viable cell count (VCC) and the specific timepoints (t).

$$IVCC_i = \int_{t=0}^{t} VCC(t)dt \approx \sum_{i=1}^{n} \frac{VCC_i + VCC_{i-1}}{2} \times (t_i - t_{i-1})$$

Equation S1: Calculation for integral of viable cell count (IVCC).

For calculation of the viable cell volume firstly the daily specific cell volume (VCV) needs to be determined by Equation S2 including the cellular diameter (d_{cell}). Subsequent, the similar calculation for IVCC determination was utilized (Equation S3).

$$VCV = \frac{4}{3} \times \pi \times VCC \times \left(\frac{d^{cell}}{2}\right)^3$$

Equation S2: Calculation for the specific volume (VCV)

$$IVCV_i = \int_{t=0}^{t} VCV(t)dt \approx \sum_{i=1}^{n} \frac{VCV_i + VCV_{i-1}}{2} \times (t_i - t_{i-1})$$

Equation S3: Integral of viable cell volume (IVCV) over the course of cultivation

The cell specific productivity (Qp) was calculated by the Equation S4 including the final mAb titer (c) and the VCC overtime.

$$qP = \frac{c_i + c_{i-1}}{t_i - t_{i-1}} \times \left(\frac{VCC_i + VCC_{i-1}}{t_i - t_{i-1}}\right)^{-1}$$

Equation S4: Calculation of the cell specific productivity (qP) over the course of cultivation.

The cell volume specific productivity (Qp_c) was calculated by the Equation S5 including the final mAb titer (c) and the VCV overtime.

$$Qp_c = \frac{c_i + c_{i-1}}{t_i - t_{i-1}} \times \left(\frac{VCV_i + VCV_{i-1}}{t_i - t_{i-1}}\right)^{-1}$$

Equation S5: Calculation of the cell specific productivity (Qp_c) over the course of cultivation.
